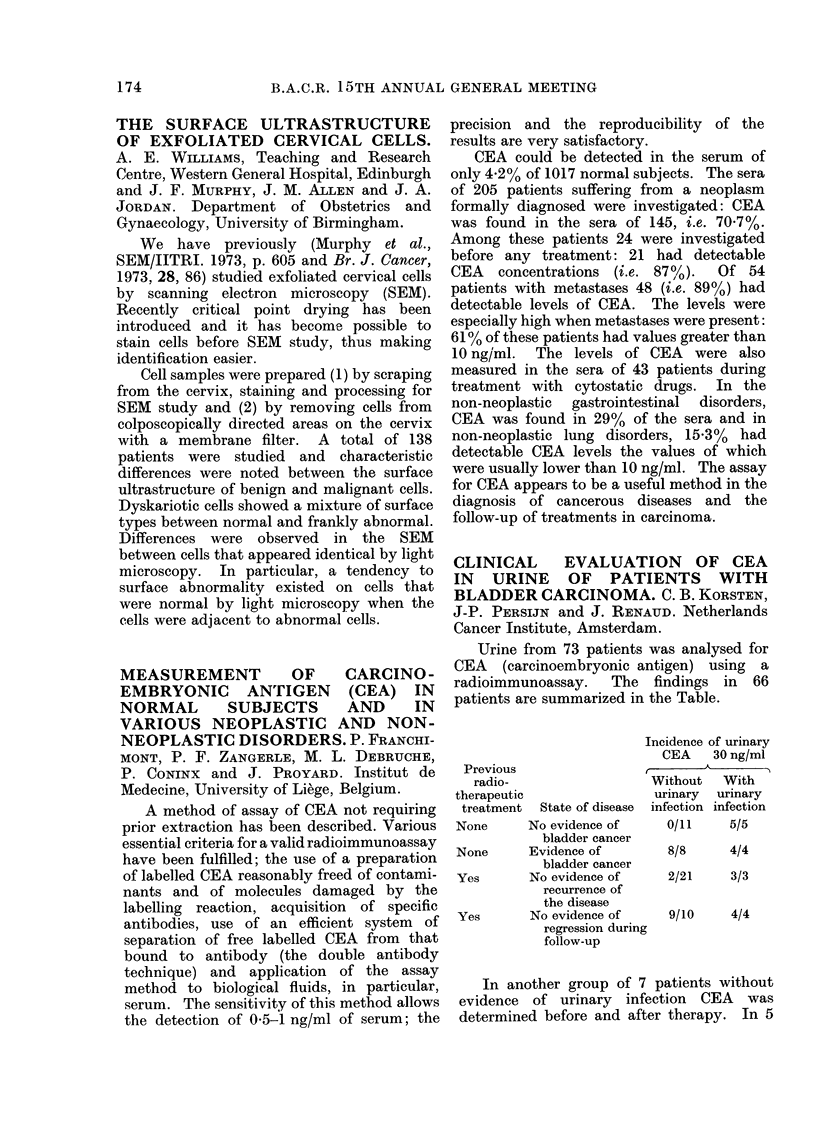# Proceedings: The surface ultrastructure of exfoliated cervical cells.

**DOI:** 10.1038/bjc.1974.135

**Published:** 1974-08

**Authors:** A. E. Williams, J. F. Murphy, J. M. Allen, J. A. Jordan


**THE SURFACE ULTRASTRUCTURE OF EXFOLIATED CERVICAL CELLS.** A. E. WILLIAMS, Teaching and Research Centre, Western General Hospital, Edinburgh and J. F. MURPHY, J. M. ALLEN and J. A. JORDAN. Department of Obstetrics and Gynaecology, University of Birmingham.

We have previously (Murphy *et al.*, SEM/IITRI. 1973, p. 605 and *Br. J. Cancer*, 1973, **28**, 86) studied exfoliated cervical cells by scanning electron microscopy (SEM). Recently critical point drying has been introduced and it has become possible to stain cells before SEM study, thus making identification easier.

Cell samples were prepared (1) by scraping from the cervix, staining and processing for SEM study and (2) by removing cells from colposcopically directed areas on the cervix with a membrane filter. A total of 138 patients were studied and characteristic differences were noted between the surface ultrastructure of benign and malignant cells. Dyskariotic cells showed a mixture of surface types between normal and frankly abnormal. Differences were observed in the SEM between cells that appeared identical by light microscopy. In particular, a tendency to surface abnormality existed on cells that were normal by light microscopy when the cells were adjacent to abnormal cells.